# Anti-factor Xa Monitoring and Activated Charcoal for a Pediatric Patient With Rivaroxaban Overdose

**DOI:** 10.5811/cpcem.2018.5.38373

**Published:** 2018-07-16

**Authors:** Brendan M. Carr, David J. Roy, Stacey A. Bangh, Thomas R. Hellmich, Laura E. Walker

**Affiliations:** * Mayo Clinic, Department of Emergency Medicine, Rochester, Minnesota; † Mayo Clinic, Pharmacy Services, Rochester, Minnesota; ‡ Hennepin County Medical Center, Minneapolis, Minnesota; § Minnesota Poison Control System, Minneapolis, Minnesota

## Abstract

Rivaroxaban, an oral anticoagulant, directly inhibits factor Xa (FXa). A 35-month-old boy was brought to the emergency department 15 minutes after ingesting 200 mg of rivaroxaban (16 mg/kg). Activated charcoal (AC) was administered; the patient was observed with monitoring of plasma anti-FXa levels and discharged the following day after an uneventful hospital observation. We identified two case series and seven case reports of potentially toxic rivaroxaban ingestion in the literature. No serious adverse effects were reported. The present case is the first reported use of anti-FXa monitoring after rivaroxaban ingestion. The magnitude of the effect of AC administration in this patient is unclear.

## INTRODUCTION

Rivaroxaban is an oral anticoagulant that directly inhibits factor Xa (FXa). The United States (U.S.) Food and Drug Administration (FDA) has approved its use in adults for prophylaxis and treatment of deep vein thrombosis, pulmonary embolism, and prevention of ischemic stroke in patients with nonvalvular atrial fibrillation. Typical daily doses in adults range from 10 to 30 mg depending on renal function and indication. No FDA-approved indications exist for pediatric patients. A favorable pharmaco-kinetic profile and absence of therapeutic drug monitoring have led to increasing popularity of rivaroxaban over vitamin K antagonists.

Until recently, no antidote was available in the U.S. to reverse the anticoagulant effects of rivaroxaban in an overdose. In May 2018, the FDA approved coagulation FXa (recombinant), inactivated-zhzo for reversal of anticoagulation in patients treated with rivaroxaban or apixaban who are experiencing life-threatening or uncontrolled bleeding. It is not yet widely available for clinical use, however, and it has not been studied in pediatric populations.^1^

Anti-FXa assays quantitatively measure plasma levels of unfractionated heparin or low-molecular-weight heparin.^2^

Multiple, commercially available, automated assays exist, and the test is readily available throughout the U.S. Compared with activated partial thromboplastin time (aPTT), a more traditional method of measuring response to heparin therapy, anti-FXa assays provide a more specific measurement of heparin activity. Other proposed advantages include faster time to achieving a therapeutic range for heparin anticoagulation, less variability in testing reagents, and fewer laboratory blood samples drawn compared with the aPTT. Anti-FXa is finalized in a similar amount of time and has been shown to be cost neutral compared with aPTT.^3^ Although there are no FDA-approved anti-FXa reagents for oral FXa inhibitors, a strong correlation has been shown with rivaroxaban concentrations using a heparin-calibrated anti-FXa assay.^1^

## CASE REPORT

A previously healthy 35-month-old boy (weight, 12.5 kg) was brought to the emergency department (ED) immediately after he was found with partially chewed rivaroxaban tablets in his mouth. His mother reported missing 10 20-mg tablets (200 mg total; approximately 16 mg/kg). The patient had no known family history of bleeding or hypercoagulable disorders.

He was examined within 15 minutes of ingestion by a physician who did not find evidence of bleeding, bruising, or altered mental status. The regional poison control center was then quickly contacted. Activated charcoal (AC) (2 g/kg) was orally administered within 45 minutes of ingestion and was tolerated well by the patient. During the ED stay, a plasma anti-FXa level was obtained approximately four hours after ingestion. The result (>4.00 international units/mL) exceeded the upper limit of the reference range and markedly surpassed the therapeutic window for unfractionated heparin (0.30–0.70 international units/mL).

The patient was admitted and observed overnight. At 13.5 hours after ingestion (a time chosen to correspond with the pediatric hospital service’s morning rounds the following day), the anti-FXa level was rechecked and found to be 1.51 international units/mL. No other laboratory testing was performed by the ED or inpatient teams. The patient was discharged later that day, less than 24 hours after ingestion, without any complications. He did not receive blood products, reversal agents, or additional doses of AC during his stay.

We performed a literature search to identify case reports of rivaroxaban ingestion. All reports of pediatric ingestion are limited to pediatric subsets of two case series drawn from reports to poison control centers with limited details for individual cases. No reports of quantitative monitoring with anti-FXa levels or utility of AC in pediatric patients were identified.

In one case series, two “1.5-year-old” children accidently ingested an unknown quantity of rivaroxaban but did not have further evaluation by a healthcare provider. Both patients were lost to follow-up without any treatment or adverse effects reported.^4^ The other case series identified 18 reports of one-time exposure in pediatric patients (age <12 years) who did not have adverse effects. An unspecified minority of patients had results of coagulation studies (international normalized ratio [INR], prothrombin time [PT], or partial thromboplastin time) that were all within the reference ranges.^5^

The other case reports, which involved adults, are summarized in the Table.^6^–^12^ AC or prothrombin complex concentrate or both were given empirically in some cases with no report of serious morbidity.

## DISCUSSION

We report the use of serum anti-FXa, a more specific marker of anticoagulation status than PT/INR in FXa inhibitors, as a means to guide management of rivaroxaban ingestion in a pediatric patient. For our patient, the true peak anti-FXa drawn at four hours was unknown, but the result (>4.00 international units/mL) exceeded the upper limit of the reference range. The anti-FXa level 13.5 hours after ingestion decreased to 1.51 international units/mL.

In healthy adult volunteers, maximal inhibition of FXa with rivaroxaban occurs in two to four hours. The bioavailability (66%–100%) is dependent on the dose and state of fasting.^13^ In healthy adults, a single rivaroxaban tablet has a half-life of six to seven hours. A dose-dependent relationship between the biologic effect and anti-FXa activity has been described.^14^

For our patient, if the most conservative peak concentration is used (4.00 international units/mL), we observed a 62% reduction in anti-FXa activity over 9.5 hours. It is plausible that AC helped decrease the absorption and bioavailability of rivaroxaban. Administration of AC within two hours after ingestion has been shown to decrease the serum concentration of rivaroxaban relatively quickly, resulting in a 43% reduction over time in the rivaroxaban area under the curve.^15^

**CPC-EM Capsule**

What do we already know about this clinical entity?

Rivaroxaban is an anti-factor Xa (FXa) inhibitor used for anticoagulation, and an overdose could be associated with bleeding. Reports of this are rare in adults and almost nonexistent in children.

What makes this presentation of disease reportable?

This is the first case report of a pediatric rivaroxaban ingestion presented in the literature and the first report of anti-FXa testing being used to confirm ingestion and monitor recovery.

What is the major learning point?

Morbidity from rivaroxaban overdose is rare. Also, anti-FXa is a common laboratory test that can be useful in cases of suspected ingestion, in lieu of traditional coagulation studies.

How might this improve emergency medicine practice?

Anti-FXa testing can be considered in diagnosing and treating a suspected rivaroxaban overdose.

It is plausible to attribute the decrease in anti-FXa in our patient to the effect of AC and possible enterohepatic recirculation of rivaroxaban. No evidence is available on the enterohepatic recirculation of rivaroxaban; however, it has been described in animal studies for apixaban and edoxaban, which are in the same therapeutic drug class as rivaroxaban.^16^,^17^ One study suggested that AC given eight hours after ingestion decreased the rivaroxaban area under the curve even after drug absorption was complete.^12^ No conclusions can be made regarding the influence of AC on our patient’s outcome given the lack of data in this population. No adverse effects related to AC administration were observed.

## CONCLUSION

In summary, our pediatric patient who accidentally ingested rivaroxaban was treated with early administration of AC and monitored with anti-FXa levels without showing clinically significant morbidity. This case showed the utility of monitoring anti-FXa levels in an ingestion of rivaroxaban and suggested that early use of AC should be considered for pediatric patients who ingest rivaroxaban. Additional pharmacokinetic and toxicokinetic studies of rivaroxaban in pediatric patients are needed to further understand optimal treatment and monitoring.

Documented patient informed consent and/or Institutional Review Board approval has been obtained and filed for publication of this case report.

## Figures and Tables

**Table:** Published case reports of rivaroxaban overdoses.

Age (years)	Sex	Time to presentation	Intentional	Amount of rivaroxaban (mg)	Coingestants	Tests performed	Treatment	Bleeding	Reference
71	M	Unknown	Yes	1,940	None	PT 60.2 s; INR 7.2; aPTT 55.7 s; BUN 28 mg/dL; Cr 1.2 mg/dL	None	None	Repplinger et al^6^
50s	M	12 h^a^	No	300a	None	INR about 2.1; PT 20 s; PTT 40 s	Activated charcoal	None	Sajkov and Gallus^7^
28	F	3 mo	Yes	Unknown	Unknown	PT 19.2 s; INR 1.8; PTT 52 s; TEG “normal”	Unknown	Abnormal uterine bleeding	Katragadda et al^8^
42	M	5 h	Yes	1,400	24 g acetaminophen; 1,200 mg codeine; 600 mg diphenhydramine; 8 mg lorazepam; unknown amount of naproxen	INR 2.4; PTT 46 s	Tranexamic acid; 4fPCC	None	Linkins and Moffat^9^
63	M	2.5 h	Yes	1,960	90 mg diazepam; 1 g quetiapine; 50 mg zolpidem	PT 66 s; aPTT 64 s	Activated charcoal; 4fPCC	None	Lehmann et al^10^
23	M	12 h	Yes	1,960	31.5 mg phenprocoumon; 1,425 mg diclofenac; 21 g metamizole	PT 34 s; aPTT 128 s	Vitamin K; PCC^b^; pantoprazole	Single episode of gross hematuria	Pfeiffer et al^11^
54	M	3 h	Yes	1,800	1,800 mg enoxaparin	PT 21.4 s; aPTT >150 s; INR 1.9	None	None	Bandali et al^12^

*aPTT*, activated partial thromboplastin time; *BUN*, blood urea nitrogen; *Cr*, creatinine; *4fPCC*, four-factor prothrombin complex concentrate; *F*, female; *g*, grams; *h*, hours; *INR*, international normalized ratio; *M*, male; *mg*, milligrams; *mo*, months; *PCC*, prothrombin complex concentrate; *PT*, prothrombin time; *PTT*, partial thromboplastin time; *s*, seconds; *TEG*, thrombolastography; *y*, years.

a Two 150-mg doses separated by 12 hours; recognized 10 minutes after the second dose.

b Case report did not specify whether three-factor or four-factor PCC was used.
